# Evaluating the Persian versions of two psoriatic arthritis screening questionnaires early arthritis for psoriatic patients questionnaire (EARP) and psoriasis epidemiology screening tool (PEST) in Iranian psoriatic patients

**DOI:** 10.3906/sag-2006-372

**Published:** 2021-02-26

**Authors:** Vahide LAJEVARDI, Zahra GHODSI, Mahdieh SHAFIEI, Amir TEIMOURPOUR, Ifa ETESAMI

**Affiliations:** 1 Department of Dermatology, Faculty of Medicine, Tehran University of Medical Sciences, Tehran Iran; 2 Department of Epidemiology and biostatistics, Faculty of Public health, Tehran University of Medical Sciences, Tehran Iran

**Keywords:** Arthritis, psoriatic, screening, psoriasis epidemiology screening tool, early arthritis for psoriatic patients questionnaire

## Abstract

**Background/aim:**

The majority of psoriatic arthritis (PsA) patients present at dermatology clinics with cutaneous psoriasis up to 10 years prior to arthritis onset; therefore, applying a suitable screening tool to detect PsA early is essential for dermatologists. This study aimed to validate and evaluate the Persian version of two PsA screening questionnaires, the early arthritis for psoriatic patients questionnaire (EARP) and the psoriasis epidemiology screening tool (PEST) in Iranian psoriatic patients.

**Materials and methods:**

In this cross-sectional study, psoriatic patients who presented to the dermatology clinic without a previously established PsA were asked to fill out the Persian version of EARP and PEST. PsA was diagnosed by a rheumatologist based on the fulfillment of the classification criteria for psoriatic arthritis. Receiver operator characteristic (ROC) curves, sensitivity, and specificity were calculated for both questionnaires.

**Results:**

A total of 75 patients (33 [44%] female, 42 [56%] male, with a mean age of 43.2 ± 14.6) were enrolled in the study. The prevalence of PsA based on rheumatologist diagnosis was 25.3% (19 patients had PsA). The ROC curve analysis of EARP and PEST were 0.949 (95% CI: 0.897–1) and 0.922 (95% CI: 0.834–1). The sensitivity of EARP and PEST questionnaires was 94.7% and 58%, respectively, while the specificity was 78.6% and 96.4%, respectively, with a cut-off of 3.

**Conclusion:**

The Persian version of both questionnaires showed good performance. We suggest EARP as a screening tool for PsA in the dermatology clinics due to much higher sensitivity with acceptable specificity compared to PEST.

## 1. Introduction 

Psoriatic arthritis (PsA) is an inflammatory skeletal disease associated with cutaneous psoriasis. Approximately 20%–30% of psoriasis patients will develop PsA during their disease course [1]. Delayed PsA diagnosis has been shown to be associated with impaired physical function, more peripheral joint erosions, and more severe axial joints involvement [2]. Therefore, early PsA detection is important to prevent irreversible joint damage.

Of note, the majority of PsA patients present at dermatology clinics with cutaneous psoriasis up to 10 years prior to arthritis onset [3]. Therefore, dermatologists play a key role in the early detection of PsA. Although various screening tools have been developed to help early PsA detection and referral to rheumatologists, a highly sensitive screening tool with acceptable specificity is vital for dermatologists. 

To classify PsA in the early stages, the group for research and assessment of psoriasis and psoriatic arthritis has developed the classification criteria for PsA (CASPAR) [4]. The currently used screening tools in psoriatic patients are psoriatic arthritis screening and evaluation (PASE) (15 questions), the psoriasis epidemiology screening tool (PEST) (5 questions), and the early arthritis for psoriatic patients questionnaire (EARP) (10 items) [5–7]. Based on different studies, PASE, PEST, and EARP have been shown to have a wide range of sensitivities and specificities, 24%–90% and 40%–94%, respectively. This wide range might be explained by various skeletal involvements in different studies or ethnic variabilities [8]. 

The pattern of musculoskeletal involvement in PsA varies among ethnic groups [3]. For example, spinal involvement has been reported more frequently in Asian patients [9]. 

The present study was conducted in consideration of the ethnic variability, the necessity of choosing a sensitive and specific screening tool in dermatology clinics, and the lack of a verified screening tool in the Persian language. The study aimed to validate the Persian version of PEST and EARP and to evaluate the sensitivity and specificity of PEST and EARP questionnaires in diagnosing PsA in Iranian patients with psoriasis (Pso) with CASPAR as the gold standard.

## 2. Materials and methods 

The present cross-sectional study was held at the psoriasis clinic at Razi dermatology hospital, Tehran University of medical sciences, between March 2019 and March 2020. The inclusion criteria were cutaneous psoriasis patients older than 18 years for whom PsA had not been diagnosed before. Informed consent was obtained from all patients. The study was approved by the Tehran University of Medical Sciences ethics committee. 

EARP [7] and PEST [6] were translated from English into the Persian language based on Beaton et al. steps [10]. First, the questionnaires were translated from English to Persian by two independent bilingual Persian speaking translators. Second, the discrepancies were resolved by a consensus between two dermatologists and the translators. Third, a back-translation was performed from Persian to English by two other bilingual translators. The back-translation versions were compared to the original English language version by a committee consisting of three dermatologists and the four translators. The discrepancies were resolved and prefinal Persian versions were developed. Twenty patients, 18 years or older, were asked to answer the prefinal Persian versions. The patients were asked to explain their problems in answering the prefinal versions. The Persian versions of EARP and PEST were finalized after resolving patients’ pretesting problems. 

Psoriatic patients without any history of PsA referred to psoriatic clinics were enrolled in the study. Clinical demographic data were recorded including age, sex, disease duration, PASI score, and nail involvement. The diagnosis of PsA was made by a rheumatologist based on the CASPAR criteria.

### 2.1. Statistical analysis 

An independent two-sample t-test was used to compare two quantitative variables. The association between two qualitative samples was evaluated by the chi-square test, and if needed, nonparametric tests, including the Mann–Whitney test and the Fisher exact test, were used. Two approaches were used for evaluating the performance of EARP and PEST questionnaires in detecting arthritis in psoriatic patients. First, multivariate logistic regression was applied. In this approach, the questions of each questionnaire (EAPR AND PEST) were considered as predictors of having arthritis compared to the rheumatologist diagnosis of arthritis as the gold standard. The performance of logistic regression in predicting patients’ arthritis status was determined by using the AUC (area under the curve) index obtained from the receiver operator characteristic (ROC) curve and its corresponding cutoff value calculated based on Youden index for each questionnaire. In the second approach, the sum of each questionnaire score was measured and the AUC index was calculated by considering this score as a continuous variable and patients’ arthritis status based on rheumatologist diagnosis as the gold standard in ROC analysis; then the value of 3 was considered as a standard cut off value. In both approaches, we reported sensitivity and specificity. All statistical analysis was performed in SPSS software (version 25, IBM Corp., Armonk, NY, USA). In this study, we considered 0.05 as a statistically significant level. The Kappa coefficient was used to evaluate the agreement between the gold standard rheumatologist diagnosis and questionnaires’ scores. 

## 3. Results 

A total of 75 patients (33 [44%] female, 42 [56%] male, with a mean age of 43.2 ± 14.6) were enrolled in the study. The prevalence of PsA based on rheumatologist diagnosis was 25.3% (19 patients). The demographic data of patients with and without arthritis are shown in Table 1. 

**Table 1:** Demographic characteristics of psoriasis patients with and without arthritis. N/A: not applicable; PsA: psoriatic arthritis; SD standard deviation.

		PsA (19)	No PsA (56)	P-value
Sex (Female/Male)		14/5	19/37	0.003
Age, years (SD)		46.79 (12.97)	41.98 (16.23)	0.246
Duration of psoriasis		12.47 (11.82)	13.05 (11.3)	0.728
Age of disease onset		34.2 (17.5)	29.63 (17.1)	0.324
PASI score (SD)		5.72 (6.1)	3.2 (3.8)	0.179
Smoking pack year (SD)		1.00 (3.45)	2.5 (5.51)	0.395
Psoriatic nail	YesNo	8 (42.1%)11 (57.9%)	16 (28.5%)40 (71.4%)	0.274
Scalp psoriasis	YesNo	10 (52.6%)9 (47.3%)	17 (30.3%)39 (69.7%)	0.08
Flexural psoriasis (%)	YesNo	8 (42.1%)11 (57.9%)	27 (48.2%)29 (51.8%)	0.64
Family history	YesNo	14 (73.7%)5 (26.3%)	32 (57.1%)24 (42.8%)	0.201
Axial involvement	YesNo	7 (36.8%)12 (63.2%)	N/AN/A	N/A
Polyarticular Oligoarticular		6 (31.6%)13 (68.4%)	N/AN/A	N/A

The internal consistency of items from the Persian version of EARP and PEST was good (Cronbach’s alpha coefficients were 0.794 [95% CI: 0.73–0.86] and 0.645 [95% CI: 0.53–0.76], respectively).

The sensitivity, specificity, positive predictive value (PPV), and negative predictive value (NPPV) were calculated for each question of the two questionnaires (Table 2). Considering EARP, the question with the highest sensitivity (100%) and NPPV (100%) was question 1 (Q1), asking about joint hurting, the question with maximum specificity (100%) and PPV (100%) was Q7, asking on the swelling or hurting of fingers for more than 3 days. For the PEST analysis, the question with maximum sensitivity (73.6) was Q1, asking on ever having a completely swollen joint; the question with maximum specificity (100%) and PPV (100%) was Q5, asking about having a swollen finger or toe for no apparent reason and the questions with maximum NPPV were Q1 and Q5, both with NPPV of 87.5%.

**Table 2:** The sensitivity, specificity, positive predictive value (PPV), and negative predictive values (NPPV) calculated for questions in the EARP and PEST questionnaires. PsA: psoriatic arthritis.

Questionnaire	PsA (Y/N)	No PsA (Y/N)	Sensitivity%	Specificity%	PPV%	NPPV%
EARP						
1. Do your joints hurt?	19/0	17/39	100	69.6	52.7	100
2. Have you taken anti-inflammatory more than twice a week for joint pain in the last 3 months?	8/11	6/50	42.1	89.2	57.1	81.9
3. Do you wake up at night because of low back pain?	7/12	5/51	36.8	91	58.3	80.9
4. Do you feel stiffness in your hands for more than 30 min in the morning?	10/9	5/51	52.6	91	66.6	85
5. Do your wrists and fingers hurt?	12/7	9/47	63.1	83.9	57.1	87
6. Do your wrists and fingers swell?	8/11	2/54	42.1	96.4	30	83
7. Does one finger hurt and swell for more than 3 days?	4/15	0/56	19	100	100	78.8
8. Does your Achilles tendon swell?	1/18	2/54	5	96	66	75
9. Do your feet or ankles hurt?	13/6	12/44	68.4	78.5	52	88
10.Do your elbow or hips hurt?	3/16	3/53	15.7	94	50	76.8
PEST						
1. Have you ever had a swollen joint (or joints)?	14/5	2/54	73.6	96.4	91.5	87.5
2. Has a doctor ever told you that you have arthritis?	10/9	2/54	52.6	96.4	83.3	85.7
3. Do your fingernails or toenails have holes or pits?	7/12	5/51	36.8	91	80.9	50
4. Have you had pain in your heel?	5/14	2/54	55.5	96.4	79.4	71.4
5. Have you had a finger or toe that was completely swollen and painful for no apparent reason?	11/8	0/56	57.8	100	100	87.5

In univariate analysis, all the questions of EARP were significantly associated with PsA, except questions 8 and 10 (Table 3). In multivariate analysis of EARP, the most relevant questions to arthritis were question 6 and question 4; the adjusted odds ratios were 27.35 (CI: 2.27–329.8) and 11.8 (CI: 2.02–68.9), respectively. Regarding the PEST questionnaire, all questions were significantly associated with PsA in univariate analysis (Table 4), and the most relevant questions to PsA were questions 1 and 2; the adjusted odds ratios were 61 (CI: 4.64–802.6) and 31.38 (CI: 3.74–263.47), respectively.

**Table 3:** Univariate and multivariate analysis of the Persian version of EARP. Unadjusted odds ratio ‡Fisher exact test. *Adjusted odds ratio for Q2, Q3, Q4, Q5, Q6, Q8, Q9, Q10. **Multivariate logistic regression, Hosmer and lemeshow test (Chi-square = 3.355, df = 4, P-value = 0.5), Nagelkerke’s R square = 0.614.

Variables		Arthritis	N(%)	UAOR†(95% CI UAOR)	P-value‡	AOR* (95% CI AOR)	P-value**
Q1	NegativePositive	YesNoYesNo	17 (30.4%)39 (69.6%)19 (100%)0 (0.0%)	-	<0.001	-	-
Q2	NegativePositive	YesNoYesNo	6 (10.7%)50 (89.3%)8 (42.1%)11 (57.9%)	6.06 (1.75, 21.02)	0.005	0.631 (0.08-4.8)	0.658
Q3	NegativePositive	YesNoYesNo	5 (8.9%)51 (91.1%)7 (36.8%)12 (63.2%)	5.95 (1.61-22.02)	0.009	1.87 (0.21-16.8)	0.577
Q4	NegativePositive	YesNoYesNo	5 (8.9%)51 (91.1%)10 (52.6%)9 (47.4%)	11.33 (3.13-41.02)	<0.001	11.8 (2.02-68.9)	0.006
Q5	NegativePositive	YesNoYesNo	9 (16.1%)47 (83.9%)12 (63.2%)7 (36.8%)	8.95 (2.77-28.95)	<0.001	2.75 (0.51-14.76)	0.239
Q6	NegativePositive	YesNoYesNo	2 (3.6%)54 (96.4%)8 (42.1%)11 (57.9%)	19.64 (3.66-105.32)	<0.001	27.35 (2.27-329.8)	0.009
Q7	NegativePositive	YesNoYesNo	0 (0.0%)56 (100%)4 (21.1%)15 (78.9%)	-	0.003	-	-
Q8	NegativePositive	YesNoYesNo	2 (3.6%)54 (96.4%)1 (5.3%)18 (94.7%)	1.5 (0.13-17.54)	0.745	0.41 (0.01-21.1)	0.655
Q9	NegativePositive	YesNoYesNo	12 (21.4%)44 (78.6%)13 (68.4%)6 (31.6%)	7.94 (2.49-25.32)	<0.001	5.1 (0.82-31.5)	0.08
Q10	NegativePositive	YesNoYesNo	3 (5.4%)53 (94.6%)3 (15.8%)16 (84.2%)	3.31 (0.61-18.05)	0.166	1.81 (0.12-28.3)	0.672

**Table 4:** Univariate and multivariate analysis of the Persian version of PEST. † Unadjusted odds ratio. ‡Fisher exact test. *adjusted Odds ratio for Q1, Q2, Q3, Q4. **Multivariate logistic regression. Hosmer and lemeshow test (chi-square = 2.834, df = 2, P-value = 0.244). Nagelkerke’s R square = 0.679.

Variable		Arthritis	N(%)	UAOR†	95% CI UAOR	P-value‡	AOR*	95% CI AOR	P-value
Q1	NegativePositive	YesNoYesNo	1 (1.8%)55 (98.2%)10 (52.6%)9 (47.4%)	61.11	6.96, 536.88	<0.001	Ref61	Ref4.64, 802.66	0.002
Q2	NegativePositive	YesNoYesNo	2 (3.6%)54 (96.4%)10 (52.6%)9 (47.4%)	30	5.62, 160.03	<0.001	Ref31.38	Ref3.74, 263.47	0.001
Q3	NegativePositive	YesNoYesNo	5 (8.9%)51 (91.1%)6 (31.6%)13 (68.4%)	4.71	1.24, 17,87	0.025	Ref1.57	Ref0.18, 14	0.684
Q4	NegativePositive	YesNoYesNo	1 (1.8%)55 (98.2%)4 (21.1%)15 (78.9%)	14.67	1.52, 141.18	0.013	Ref20.04	Ref1.1, 364.34	0.043
Q5	NegativePositive	YesNoYesNo	21 (37.5%)35 (62.5%)19 (100%)0 (0.0%)	-	-	<0.001	-	-	-

The AUC (area under the curve) calculated based on multivariate analyses and the Youden index of EARP and PEST was 0.949 (95% CI: 0.89–1.00) and 0.922 (95% CI: 0.834–1.00), respectively (Figure). The calculated sensitivity and specificity based on this method are shown in Table 5. When the cutoff of 3 was selected for EARP and PEST based on the previous studies [7], the calculated sensitivity and specificity by ROC curve are shown in Table 5. As shown in Table 5, the sensitivity of 0.947 that was measured for EARP was higher than that of PEST at 0.58. Conversely, the specificity of EARP was lower than that of PEST (0.786 compared to 0.964).

**Figure:**
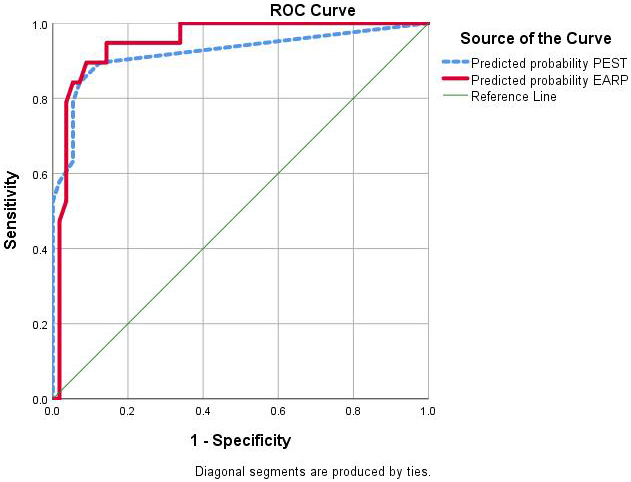
The AUC (area under the curve) calculated based on multivariate analyses and Youden index for EARP and PEST.

**Table 5:** Calculated sensitivity and specificity for EARP and PEST questionnaires with a cut off of 3 and without a predefined cut-off based on the Youden index by ROC curve analysis. AUC: area under the curve

	AUC (95% CI)	Sensitivity	Specificity	Cut-off value
EARP	0.923 (0.882-0.99)	0.947	0.786	3
PEST	0.936 ( 0.864-0.983)	0.58	0.964	3
Based on Youden-index				
EARP	0.949 (0.897-1.00)	94.7%	85.7	0.213
PEST	0.922 (0.834 – 1.00)	78.9	94.6	0.574

## 4. Discussion 

This study showed that the Persian version of EARP and PEST had an acceptable performance for screening PsA in Iranian psoriatic patients presenting to the dermatology clinic (EARP, sensitivity = 0.947 and specificity = 0.786; PEST, sensitivity = 0.58 and specificity = 0.964). The sensitivity of EARP in this study was higher than the primary validation study and other language versions validation studies, while the measured specificity was slightly lower than the previous studies [5,7]. The sensitivity of PEST in this study (58%) was much lower than in the primary validation study (92%) and other language version study (79.3%), while the measured specificity was much higher than the primary validation study (78%) and other language version study (79.3%) [5,6]. Similar to this study, Mishra et al. also reported very low sensitivity (53.3%) for the PEST questionnaire and they considered the low sensitivity as a major drawback for this questionnaire [11].

The different performances between various studies may result from different patient characteristics, including the prevalence of PsA among psoriatic patients, the pattern of articular involvement (axial versus peripheral), and ethnic variabilities. The prevalence of PsA in this study was 25.3%, whereas a range of 12.9% to 78.6% of PsA was reported in various studies evaluating the PsA screening tools [5–7]. In this study, patients without a previous diagnosis of PsA were included, whereas other studies enrolled patients with both established diagnosis of PsA and newly diagnosed ones.

The difference in the pattern of articular involvement may also explain the variation in screening tool performances. The majority of current PsA screening tools evaluate peripheral arthritis more than axial arthritis; for example, some studies showed that PEST missed a high proportion of axial involvement and enthesitis [12]. In this study, vertebral and iliac involvement was much lower than in Chiowchanwisawakit et al.’s study (36.8% compared to 56%, respectively), which may be responsible for the different performances of PEST in the two studies [5]. However, in this study, the performance of the questionnaires was not assessed in subgroups of patients with different patterns of articular involvement (including axial, polyarticular, and oligoarticular) due to the low number of patients in each subgroup. This limitation could have affected the sensitivity and specificity measured in this study.

The question most associated with arthritis in the EARP questionnaire in this study was question 6, “Do your wrists and fingers swell?”, in concordance with Chiowchanwisawakit et al.’s study that found the same question to be the most relevant question to arthritis [5]. The most relevant question to arthritis in the PEST questionnaire was question 1, “Have you ever had a swollen joint (or joints)?” and the second most relevant question was question 2, “Has a doctor ever told you that you have arthritis?” in this study, similar to Ibrahim et al.’s study that reported these two questions as the most significant predictors of arthritis in psoriatic patients [6]. Therefore, these questions can be most helpful for deciding whether to refer patients to rheumatologists.

Selecting an optimized screening tool depends on the patients’ characteristics; in this study, the sensitivity of PEST (58%) was lower than that of EARP (94.7%). Considering the prevalence of nearly 20%–30% of PsA in psoriatic patients presenting to the dermatology clinic, a screening test with high sensitivity and medium specificity seems ideal to ensure no cases of true PsA are missed and also not over referring psoriatic patients to rheumatology clinics as well. Therefore, in the Iranian population of psoriatic patients, EARP is suggested more often for screening PsA due to its higher sensitivity compared to PEST. However, in a population of general patients in whom the prevalence of PsA is much lower than psoriatic patients, the PEST questionnaire may be an ideal option with fewer questions and more simplicity of application.

The strength of this study was enrolling psoriatic patients without a previous history of PsA. Previous studies that included both diagnosed and undiagnosed PsA might have overestimated the sensitivity due to recall bias in patients with established PsA [5,6].

In summary, both EARP and PEST questionnaires showed acceptable performances in the Iranian psoriatic population without a previously established diagnosis of psoriatic arthritis (sensitivity 94.7% and 58%, specificity 78.6% and 96.4%, respectively). The most relevant questions in both questionnaires were questions asking about the swelling of joints. Due to the higher sensitivity calculated for EARp compared to PEST in this study, we suggest applying EARP for screening psoriatic arthritis in psoriatic patients with a higher prevalence of arthritis compared to the general population.

## Informed consent

Informed consent was obtained from all patients. This study was approved by the Tehran University of Medical Sciences ethics committee.
